# Calorie Restriction Suppresses Age-Dependent Hippocampal Transcriptional Signatures

**DOI:** 10.1371/journal.pone.0133923

**Published:** 2015-07-29

**Authors:** Marissa J. Schafer, Igor Dolgalev, Melissa J. Alldred, Adriana Heguy, Stephen D. Ginsberg

**Affiliations:** 1 Cell and Molecular Biology Program, New York University Langone Medical Center, New York, New York, United States of America; 2 Center for Dementa Research, Nathan Kline Institute, Orangeburg, New York, United States of America; 3 Genome Technology Center, New York University Langone Medical Center, New York, New York, United States of America; 4 Department of Psychiatry, New York University Langone Medical Center, New York, New York, United States of America; 5 Department of Pathology, New York University Langone Medical Center, New York, New York, United States of America; 6 Department of Neuroscience and Physiology, New York University Langone Medical Center, New York, New York, United States of America; University of Wisconsin, UNITED STATES

## Abstract

Calorie restriction (CR) enhances longevity and mitigates aging phenotypes in numerous species. Physiological responses to CR are cell-type specific and variable throughout the lifespan. However, the mosaic of molecular changes responsible for CR benefits remains unclear, particularly in brain regions susceptible to deterioration during aging. We examined the influence of long-term CR on the CA1 hippocampal region, a key learning and memory brain area that is vulnerable to age-related pathologies, such as Alzheimer’s disease (AD). Through mRNA sequencing and NanoString nCounter analysis, we demonstrate that one year of CR feeding suppresses age-dependent signatures of 882 genes functionally associated with synaptic transmission-related pathways, including calcium signaling, long-term potentiation (LTP), and Creb signaling in wild-type mice. By comparing the influence of CR on hippocampal CA1 region transcriptional profiles at younger-adult (5 months, 2.5 months of feeding) and older-adult (15 months, 12.5 months of feeding) timepoints, we identify conserved upregulation of proteome quality control and calcium buffering genes, including heat shock 70 kDa protein 1b (Hspa1b) and heat shock 70 kDa protein 5 (Hspa5), protein disulfide isomerase family A member 4 (Pdia4) and protein disulfide isomerase family A member 6 (Pdia6), and calreticulin (Calr). Expression levels of putative neuroprotective factors, klotho (Kl) and transthyretin (Ttr), are also elevated by CR in adulthood, although the global CR-specific expression profiles at younger and older timepoints are highly divergent. At a previously unachieved resolution, our results demonstrate conserved activation of neuroprotective gene signatures and broad CR-suppression of age-dependent hippocampal CA1 region expression changes, indicating that CR functionally maintains a more youthful transcriptional state within the hippocampal CA1 sector.

## Introduction

Dysfunctional synaptic connections and neurodegeneration are posited to be the cellular origins of age-dependent memory and cognitive impairment [1]. The hippocampal formation, in particular the CA1 hippocampal sector, is a central learning and memory hub within the mammalian brain that displays activity-dependent synaptic plasticity in neural network formation [2]. CA1 pyramidal neurons are severely affected in AD, while several other temporal lobe and hippocampal cell types are relatively spared throughout pathology progression. The compilation of cellular processes responsible for this selective vulnerability are not fully understood [3,4].

The hippocampal region is prone to abnormal protein aggregation, suggesting proteome quality control dysfunction throughout aging [1]. In normal hippocampal aging, characterized by *ad libitum* (AL) feeding and no overt pathology, spatial memory deficits coincide with downregulation of genes involved in the unfolded protein response, including heat shock 70 kDa protein 5 (Hspa5) and calreticulin (Calr) [5], as well as negative regulation of synaptic plasticity genes [6]. Intrinsic electrical and structural characteristics of CA1 pyramidal neurons may also contribute to neurodegenerative vulnerability, where susceptibility to excitotoxicity may originate from diminished calcium buffering capacity in older age, relative to less excitable cell types [7,8]. Furthermore, CA1 pyramidal neurons are dependent on pro-survival trophic factor signaling, including brain-derived neurotrophic factor (Bdnf), and reduction in trophic factor signaling throughout aging, which occurs coincident with neuron loss and memory impairment, may also contribute to the selective vulnerability phenotype [9,10].

Calorie restriction (CR) is a dietary regimen that involves a sustained, moderate reduction (typically 20–40%) in calorie intake compared to AL feeding. CR has proven to be a powerful method in multiple species to reduce the incidence of chronic disease and increases the lifespan. CR feeding dramatically alters many processes associated with dysfunctional brain aging, and serves as an essential tool for understanding endogenous attenuation of age-related pathology [11–15]. CR enhances expression of Bdnf and neurotrophin 3 (Ntf3) [11] while reducing aberrant protein aggregation [12,16], excitability [13], and calcium dysregulation [14]. Partial suppression of age-dependent gene expression changes have been observed within the neocortex and cerebellum of aged CR mice compared to AL feeding [17], and additional investigations identified a unique CR-specific transcriptional profile within the hippocampal CA1 region, relative to adjacent less vulnerable hippocampal subregions [18]. Previous investigations of changes underlying normal brain aging and CR-benefits relied on hybridization methodologies, including microarray analysis [5,17–23], which have limited profiling capacity and quality relative to total mRNA sequencing [24]. Presently, we test the hypothesis that long-term CR beneficially modifies age-dependent gene expression using unbiased total mRNA sequencing and NanoString nCounter profiling in the selectively vulnerable hippocampal CA1 region, an area implicated in memory function that is prone to age-related neurodegenerative pathology [2,25,26].

## Materials and Methods

### Mouse model and tissue accession

Animal protocols for this study were in agreement with NIH guidelines and approved by the Institutional Animal Care and Use Committee (IACUC) of the Nathan Kline Institute and NYU Langone Medical Center. A subset of the mice applied to these experiments were used as control mice in a recently published study [16]. At approximately 2.5 months of age, female Swiss Webster x DBA/C57BL6 F1 mice were randomly assigned to AL or 30% CR (reduction specific to carbohydrates) dietary regimens (AL, #D12450B; 30% CR, #D03020702B; Research Diets Inc., New Brunswick, NJ). CR mice were fed their daily allotment in the morning, and CR diets were adjusted weekly, according to the average daily intake of the AL group. Body weights were measured twice weekly. At approximately 5 or 15 months of age, following 2.5 or 12.5 months of diet administration, respectively, mice were administered a lethal dose of ketamine (80 mg/kg) and xylazine (13 mg/kg), perfused transcardially with ice-cold 0.1 M phosphate buffer, and brains were rapidly removed. Sacrifices took place in mid-afternoon. The hippocampal CA1 region was microdissected from tissue slabs using a dissecting microscope, frozen on dry ice, and stored at -80°C, as described previously [27].

### RNA isolation, library preparation, and sequencing

RNA was isolated from frozen hippocampal CA1 microdissections using the miRNAeasy Micro Kit (Qiagen, Valencia, CA) according to manufacturer specifications. Bioanalysis (2100, Agilent Biotechnologies, Santa Clara, CA) was employed to determine RNA concentration and quality. 300 ng of total RNA with an average RNA integrity number (RIN) value of 9 was applied to the TruSeq RNA Sample Preparation Kit v2 (Illumina, San Diego, CA) to construct mRNA sequencing libraries. The quality of the libraries was assessed using Agilent bioanalysis, and quantification was performed by qPCR. Libraries were applied to 8 runs of paired-end 50-base pair sequencing on a HiSeq 2500 platform (Illumina), using the rapid run mode. Five and six biological replicates were sequenced per diet condition for 5 and 15 month groups, respectively.

### Statistical analysis and functional annotation of gene expression

Base calling was performed using Illumina bcl2fastq software. The sequencing reads were aligned to the mouse genome (UCSC build mm10) using the splice-aware TopHat aligner. Likely PCR duplicates were removed with Picard MarkDuplicates tool. Filtered mapped reads were analyzed using the Cufflinks package with default parameters and the following additions. A fragment bias correction was applied to improve the accuracy of transcript abundance estimates, and a multi-read correction was applied to weight reads mapping to multiple locations in the genome. The dispersion estimation method used was default pooling, in which each replicated condition was modeled and averaged to provide a single global model for all conditions. Differentially expressed genes were determined based on false discovery rate (FDR)-adjusted p-value <0.05 (q) as calculated by Cuffdiff. Ingenuity Pathway Analysis (IPA) was used to functionally annotate datasets of differentially expressed genes (p<0.01, q<0.05).

### NanoString nCounter analysis

NanoString nCounter (NanoString Technologies, Seattle, WA) digital mRNA detection was used to validate mRNA sequencing data. A custom code set was generated to probe transcript levels of housekeeping genes, the levels of which did not change as a function of diet or age, and differentially expressed targets. 100 ng of total RNA was hybridized to capture-reporter probe sets and immobilized on NanoString cartridges, according to manufacturer specifications. Following excess RNA and probe removal, digital barcodes were counted and processed with NanoString nCounter analysis software for quality control and normalization. Counts were normalized to positive controls and the housekeeping genes as recommended by NanoString. Two-tailed t-tests were used to determine expression differences amongst conditions. Log transformed fold change values for mRNA sequencing and NanoString values were compared using Pearson product-moment correlation coefficient.

## Results

### Long-term CR stably reduces body weight

**Fig 1:**
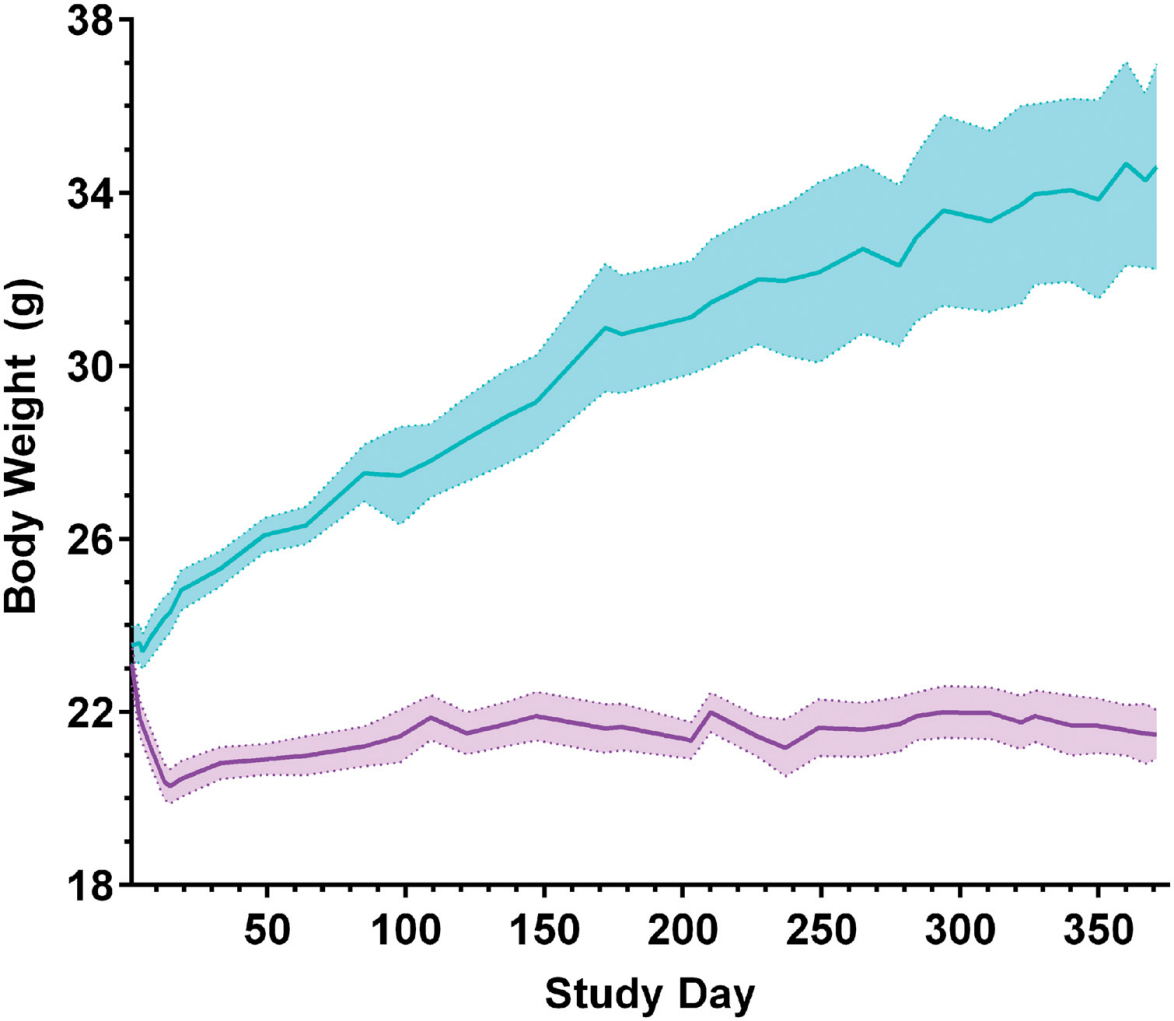
Average body weight following sustained 30% CR or AL feeding. Beginning at approximately 2.5 months of age, wild-type female mice were maintained on 30% CR (purple) or AL (blue) diets and were sacrificed following 2.5 or 12.5 months of diet administration. Body weight was measured approximately twice weekly. Within the first 2 weeks of AL and CR feeding, mice maintained on the 30% CR diet lost an average of 12% of their body weight (t-test, p<0.001), which stabilized for the remainder of the study. For study days 1–85, n = 12–18, and for study days 85–365, n = 6–14, reflecting sacrifice of the first group of mice at 5 months of age; mean +/- SEM.

Mice subjected to CR feeding typically undergo significant body weight reductions upon initial diet introduction and then stabilize to relatively constant levels, which are lower than their AL-fed counterparts [28]. At the onset of our study, body weights were not significantly different for 2.5 month old wild-type mice either randomized to AL or 30% CR cohorts. The average body weight of mice fed the continuous 30% CR diet decreased by approximately 12% (p<0.001) in the first 2 weeks of CR feeding. Thereafter, body weights of CR mice stabilized and remained constant throughout the remaining 2.5 or 12.5 months of diet administration (Fig 1).

### CR-activated gene signatures predict opposition of aging programs

**Fig 2:**
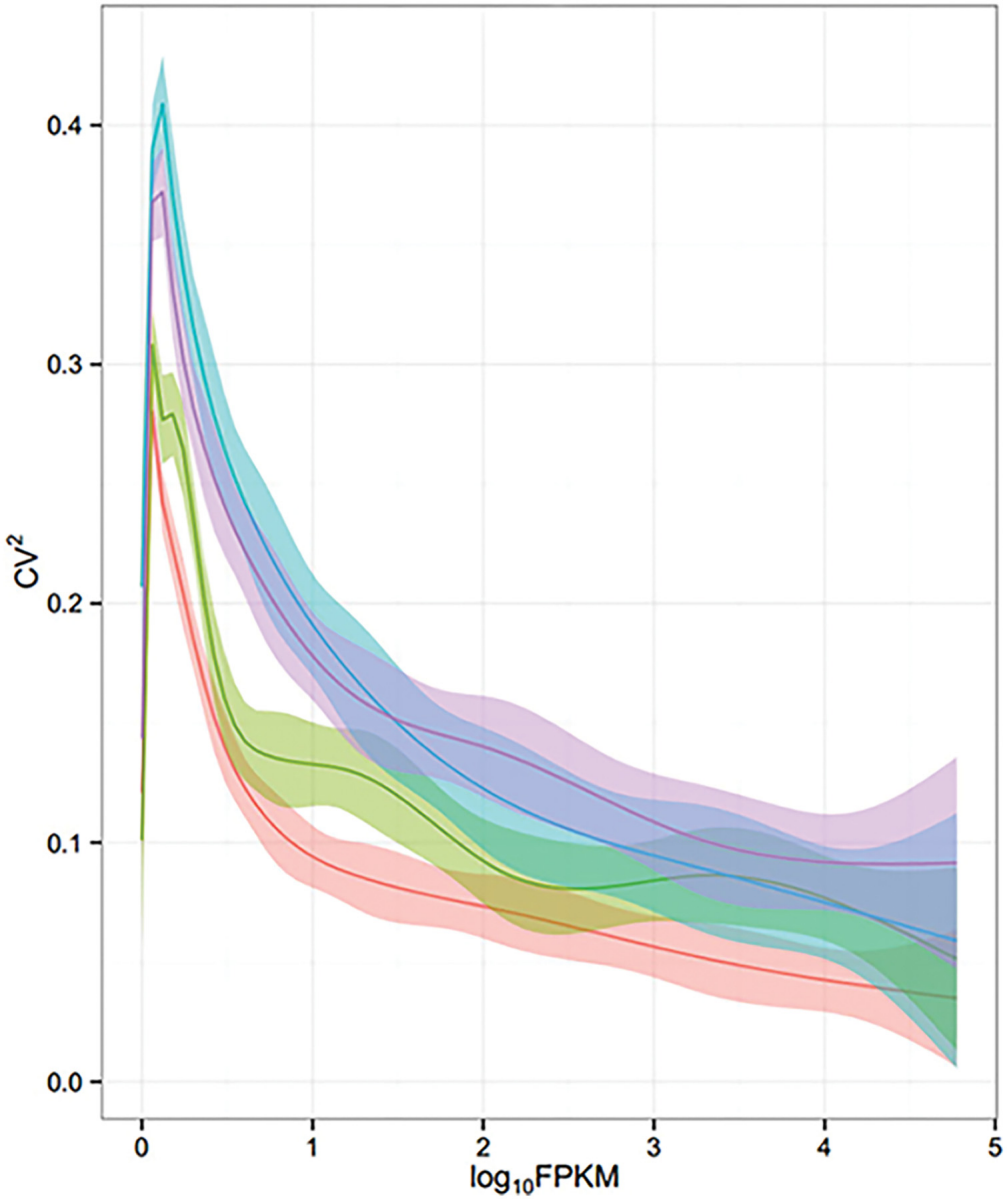
Biological variance within mRNA sequencing condition groups. The squared coefficient of variation (CV^2^) was plotted against Fragments Per Kilobase of exon per Million fragments mapped (log_10_FPKM), representing the total distribution of mRNA sequence reads for each condition group, which are depicted as follows, 5 months AL (coral), 5 months CR (green), 15 months AL (blue), and 15 months CR (purple).

Using RNA isolated from hippocampal CA1 microdissections from younger-adult (5 months, 2.5 months of feeding) and older-adult (15 months, 12.5 months of feeding) wild-type mice maintained on 30% CR or AL diets, we performed 50 nucleotide paired-end total mRNA sequencing, yielding an average of 73.6 million paired reads per sample. We observed marginally larger coefficient of variation values within the 15 month condition groups, reflective of greater variability in aged samples (Fig 2).

**Fig 3:**
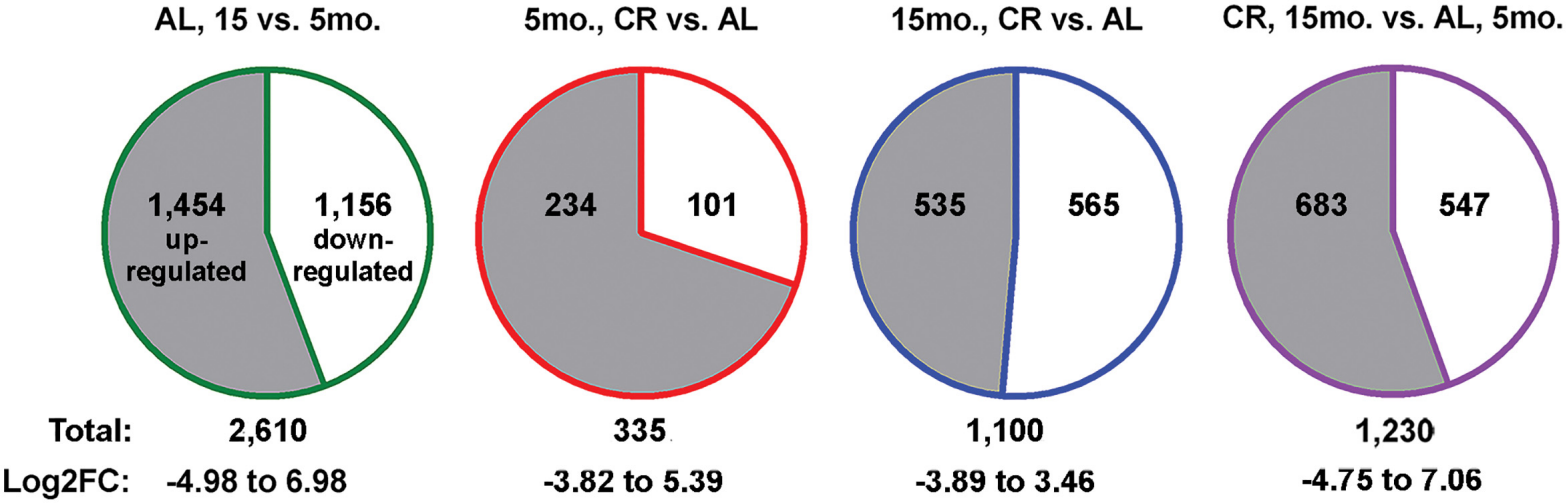
Differential gene expression via mRNA sequence analysis pairwise comparisons. The total number of differentially expressed transcripts identified by total mRNA sequencing within each pairwise comparison are indicated, along with the log_2_ fold change (Log2FC) range and the number of upregulated (gray) and downregulated (white) targets (p<0.01, q<0.05).

Comparison of CA1 expression profiles from 15 month old mice to those of 5 month old mice maintained on the control AL diet identified 2,610 differentially regulated transcripts within a log2 fold change range of -4.98 to 6.98 (p<0.01, q<0.05) with 1,454 upregulated and 1,156 downregulated genes (Fig 3 and S1 Table), representing the normal aging transcriptional signature in middle-aged adulthood. Within 15 month old mice maintained on CR vs. AL diets, we identified 535 upregulated and 565 downregulated genes for a total of 1,100 differentially regulated transcripts over a log2 fold change range of -3.89 to 3.46 (p<0.01, q<0.05) (Fig 3 and S2 Table).

**Fig 4:**
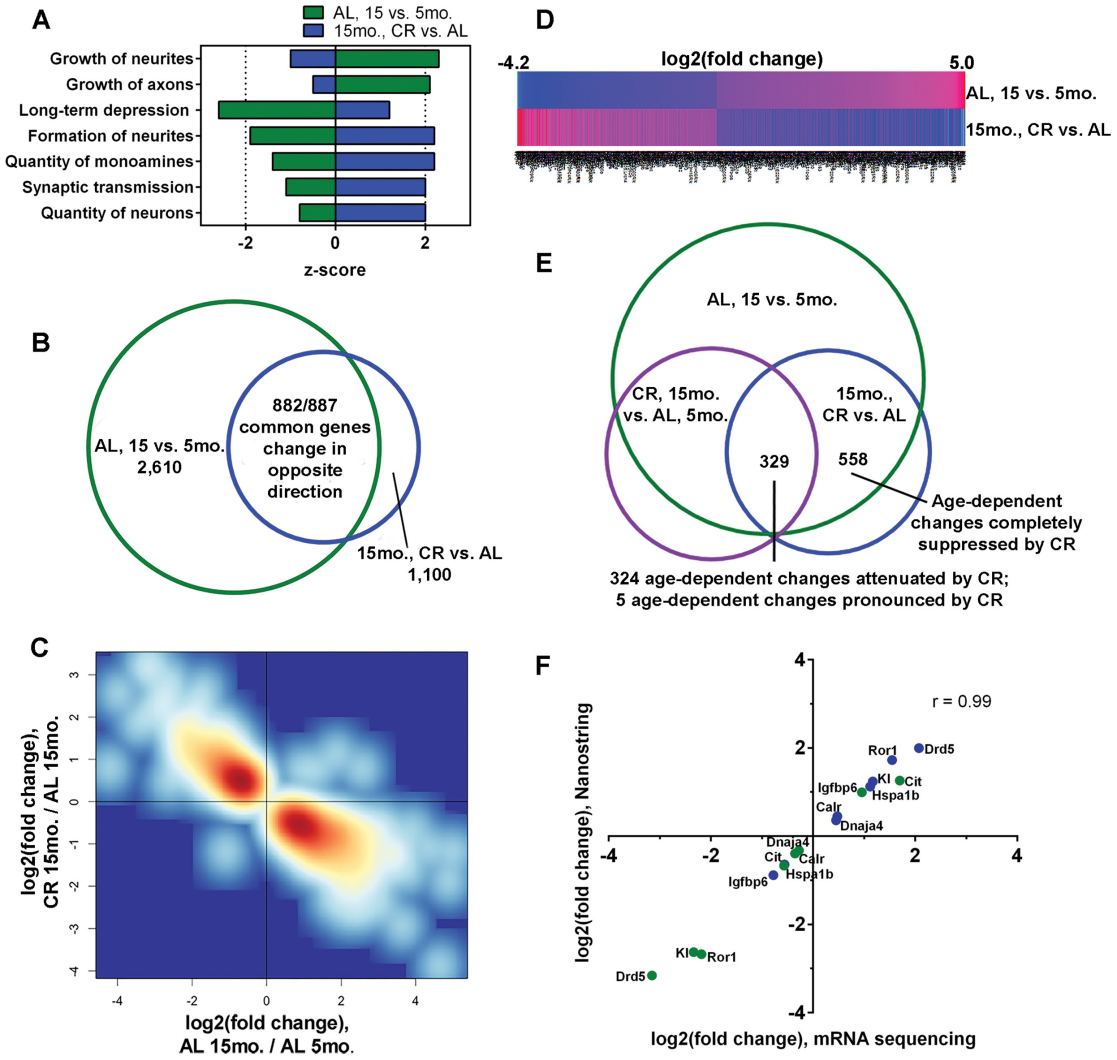
Long-term CR opposes age-dependent cellular functions and reverses age-dependent gene expression in the hippocampal CA1 sector. (A) Comparative IPA was used to predict opposing functions activated (z-score ≥ 2) or inactivated (z-score ≥ -2) within the AL, 15 vs. 5 month and 15 month, CR vs. AL datasets. (B) 882 of the 887 gene changes common to the normal aging (AL, 15 vs. 5 month) and CR diet in aged CA1 (15 month CR vs. AL) datasets occur in the opposite direction. (C) Smoothed density representation of the normal aging changes (AL 15 month/AL 5 month) versus the CR diet in aged CA1 changes (CR 15 month/AL 15 month) for the 887 genes common to both datasets, illustrating CR reversal of age-dependent changes. (D) Heatmap depiction the 882 age-dependent expression changes suppressed by CR, comparing the mean log_2_ fold change values for each gene (p<0.01, q<0.05). (E) 558 transcriptional changes identified in the AL, 15 vs. 5 month and 15 month, CR vs. AL datasets were not identified in the CR, 15 month vs. AL, 5 month dataset, indicating no significant differences in expression levels for these genes. 329 differentially expressed genes were common to all 3 profiles, and 5 of these age-dependent changes were made more pronounced by CR. For the remaining 324 genes, CR 15 month expression levels were in between AL 5 month and AL 15 month levels (p<0.01, q<0.05). (F) Correlation of log_2_ fold change levels of 8 genes assessed by mRNA sequencing (p<0.01, q<0.05) and NanoString nCounter (p<0.05); Pearson correlation value (p<0.0001) is indicated; AL, 15 vs. 5 month (green) and 15 month, CR vs. AL (blue); dopamine receptor D5 (Drd5), receptor tyrosine kinase-like orphan receptor 1 (Ror1), klotho (Kl), heat shock 70 kDa protein 1B (Hspa1b), calreticulin (Calr), DnaJ (Hsp40) homolog subfamily A member 4 (Dnaja4), insulin-like growth factor binding protein 6 (Igfbp6), citron (Cit).

Functional annotation comparisons of our total ‘normal aging’ (AL, 15 vs. 5 month; 2,610 genes) and ‘CR in aged CA1’ (15 month, CR vs. AL; 1,100 genes) datasets predicted that growth functions, including growth of axons and neurites, would be age-related and reduced by CR. Inactivation of long-term depression (LTD) was predicted in normal aging, which was opposed by CR. In the 15 month CR vs. AL dataset, CR was expected to enhance synaptic plasticity functions, including synaptic transmission and neurite formation, as well as neuron and monoamine quantity, and these CR-activation predictions were antithetical in the normal aging dataset (Fig 4A and S3 Table). Within targets significantly altered by CR at the 15 month timepoint, we observed significant upregulation of neurotrophic factors, Bdnf and Ntf3 (S2 and S3 Tables), CR-responsive targets [11] implicated in hippocampal synaptic transmission and plasticity [29].

### CR suppresses age-dependent expression changes for hundreds of transcripts

By conducting functional comparisons, we noted that long-term CR significantly reduced the magnitude of the normal age-dependent change for several genes, so we queried our datasets to determine the total number of differentially expressed genes in the normal aging dataset (AL, 15 vs. 5 month) that were also differentially expressed in the aged CR dataset (15 month, CR vs. AL). 887 of the 1,100 genes (81%) that were altered by CR in the 15 month old mice were also significantly altered by normal aging (Fig 4B). Of these 887 common genes, 99% (882 out of 887) of the age-dependent changes were reversed by CR (Fig 4B–4D and S4 Table). 558 of the 882 age-dependent changes reversed by CR were not differentially expressed upon comparison of 15 month CR expression levels to 5 month AL expression levels, indicating that these 558 age-dependent changes were prevented by CR. 15 month CR expression levels for the remaining 324 of 882 genes were in between and significantly different than both 5 month AL and 15 month AL expression levels, suggesting that CR was reducing, but not completely blocking, the age-dependent changes (Fig 4E and S4 Table). Age-dependent changes of only 5 transcripts, alanine-glyoxylate aminotransferase 2-like 1, calbindin 2, collagen type VIII alpha 2, receptor transporter protein 1, and stabilin 2, became more pronounced following CR (S5 Table), signifying minimal CR exacerbation of aging expression signatures within the hippocampal CA1 region, relative to the large cohort of aging-altered genes attenuated by CR.

To investigate the biological significance of the CR-suppressed expression profile, we functionally annotated the dataset of 882 age-dependent expression changes. Top canonical pathways enriched in the CR-suppression dataset included calcium, axonal guidance, corticotrophin-releasing hormone (Crh), G-protein coupled receptor, long-term potentiation (LTP), and Creb signaling (Table 1). We demonstrate CR-dependent blunting of age-dependent downregulation of NMDA receptor subunits NR2A and NR2B (Grin2a, Grin2b) and AMPA1 receptor subunit (Gria1) mRNA levels (Table 1 and S4 Table), changes potentially underlying previously observed LTP maintenance throughout aging [30]. In contrast, mRNA levels corresponding to stress-related Crh and its cognate receptor, corticotrophin-releasing hormone receptor 1 (Crhr1), were both significantly increased in an age-related manner, and this effect was suppressed by CR.

**Table 1:** Canonical pathways functionally associated with CR-suppressed expression signature in older-adult hippocampal CA1. (p<0.01, q<0.05).

Canonical pathway	p-value	Altered genes / Known related genes	Upregulated by CR (Downregulated in normal aging)	Downregulated by CR (Upregulated in normal aging)
Calcium signaling	7.35E-06	22/140 (16%)	Atp2b1, Camk2b, Camkk1, Chrna5, Gria1, Grin2a, Grin2b, Ppp3ca, Ryr3, Trpc4	Atp2b4, Camk1, Camk2d, Camk4, Chrna4, Chrna6, Chrnb3, Gria4, Itpr3, Mef2c, Slc8a3, Trpc3
Axonal guidance signaling	4.93E-05	38/352 (11%)	Actr2, Actr3, Arpc2, Arpc5, Epha4, Epha6, Epha7, Git1, Gnaq, Gng10, Gng7, Nrp1, Ppp3ca, Prkce, Prkcg, Sema3e, Sema5a, Slit1, Slit3, Wnt2	Bmp7, Ecel1, Efna5, Epha8, Gnb4, L1cam, Ngef, Pak7, Plcb4, Plxna3, Plxnd1, Rnd1, Sema3a, Sema3f, Sema4a, Unc5d, Wnt10a, Wnt6
Corticotropin releasing hormone signaling	2.19E-04	15/96 (16%)	Arpc5, Elk1, Gnaq, Npr1, Prkce, Prkcg	Adcy8, Camk4, Crh, Crhr1, Fos, Gucy1a3, Itpr3, Mapk11, Mef2c
Synaptic long term potentiation	3.47E-04	15/100 (15%)	Camk2b, Gnaq, Gria1, Grin2a, Grin2b, Grm1, Ppp3ca, Prkce, Prkcg	Adcy8, Camk2d, Camk4, Gria4, Itpr3, Plcb4
Creb signaling in neurons	5.12E-04	19/149 (13%)	Camk2b, Elk1, Gnaq, Gng10, Gng7, Gria1, Grin2a, Grin2b, Grm1, Prkce, Prkcg	Adcy8, Camk2d, Camk4, Gnb4, Gria4, Grik3, Itpr3, Plcb4
G-protein coupled receptor signaling	3.23E-04	26/228 (11%)	Drd5, Dusp6, Gnaq, Grm1, Htr1a, Htr4, Mc4r, Prkce, Prkcg, Ptk2b, Rasgrp1, Rgs14	Adcy8, Adra1b, Adrb3, Camk2d, Camk4, Crhr1, Dusp1, Hrh1, Htr2c, Htr7, Oprk1, Pde4d, Plcb4, Ptgdr

To validate the CR-suppressed aging gene expression signature we employed NanoString nCounter analysis, a methodology that enables multiplex digital counting of individual transcripts [31]. Nanostring nCounter profiling confirmed CR- and age-dependent expression changes for a subset of differentially expressed targets (Fig 4F). Specifically, we observed CR-dependent upregulation of dopamine receptor D5 (Drd5), receptor tyrosine kinase-like orphan receptor 1 (Ror1), klotho (Kl), heat shock 70 kDa protein 1B (Hspa1b), calreticulin (Calr), and DnaJ (Hsp40) homolog subfamily A member 4 (Dnaja4) in the hippocampal CA1 region of 15 month old mice, compared to AL feeding, and these targets were all significantly downregulated during normal aging in AL-fed mice. Conversely, we observed CR-dependent downregulation of insulin-like growth factor binding protein 6 (Igfbp6) and citron (Cit) in the hippocampal CA1 region of 15 month old mice, compared to AL feeding, and these targets were all significantly upregulated in normal aging in AL-fed mice. The log2 fold change values for mRNA sequencing and NanoString nCounter data were strongly correlated (r = 0.99), suggesting high quantitative precision within the total mRNA sequencing dataset (Fig 4F).

### Conserved neuroprotective signatures are activated within dynamically shifting aging-related CR transcriptional responses

**Fig 5:**
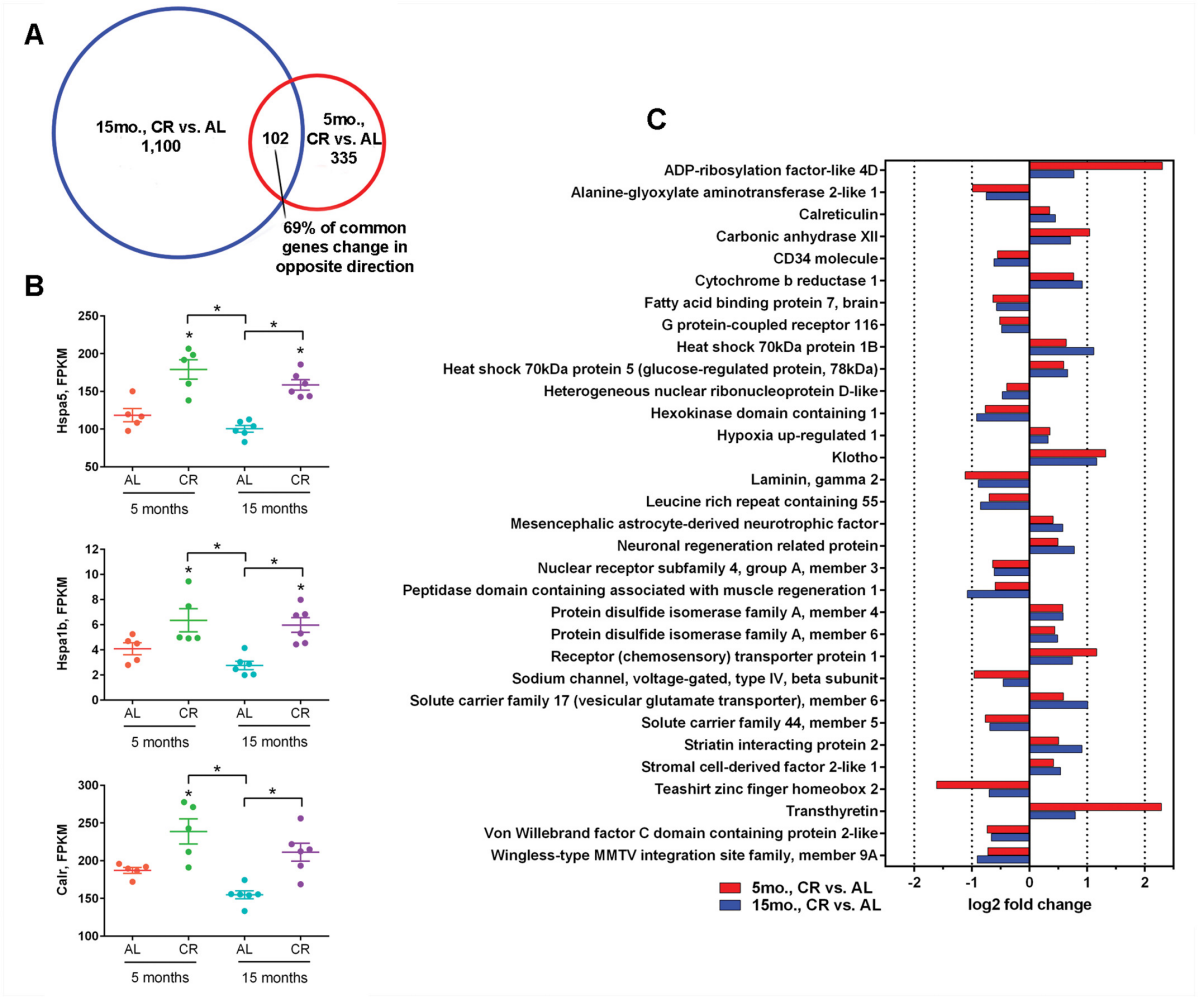
CR upregulation of neuroprotective gene signatures is conserved at 5 and 15 months, despite divergent transcriptional profiles. (A) 102 genes are common to both the 15 and 5 month CR vs. AL differential expression mRNA sequencing profiles. Of the 102 common genes, 70/102 (69%) of significant changes occur in the opposite direction. (B) Normalized age- and diet-dependent FPKM levels for select protein folding and calcium buffering genes (*p<0.01, q<0.05); heat shock 70 kDa protein 5 (Hspa5), heat shock 70 kDa protein 1B (Hspa1b), calreticulin (Calr). (C) Log_2_ fold change levels for the 32 conserved gene expression changes within 15 (blue) and 5 (red) month CR vs. AL expression profiles that occurred in the same direction are depicted (p<0.01, q<0.05).

Regarding the total number of transcripts altered within each mRNA sequencing dataset, aging more strongly influenced gene expression, relative to diet (Fig 3). Hence, we posited that CR would affect transcriptional profiles differently in younger-aged and older-aged mice, but conserved CR-signatures would be identifiable. Upon assessment of 5 month old mice maintained on the CR vs. AL diets, we identified 335 differentially expressed genes, of which, 234 transcripts were upregulated and 101 transcripts were downregulated within a log2 fold change range of -3.81 to 5.39 (p<0.01, q<0.05) (Fig 3 and S6 Table). We compared these 335 differentially expressed genes to the 1,100 expression changes identified in 15 month old mice maintained on CR vs. AL diets and identified 102 genes altered at both ages. Only 31% (32/102) of common genes were altered in the same direction (Fig 5A), suggesting that proteins encoded by CR-responsive transcripts may serve different functional roles in the hippocampus of younger-adult and older-adult brains. Within conserved changes, an enrichment of transcripts implicated in proteome quality control and calcium buffering was detected, including upregulation of Hspa5 and Calr expression (Fig 5B), downregulation of which has been associated with age-dependent hippocampal dysfunction [5], as well as increased expression of Hspa1b (Fig 5B) and protein disulfide isomerase family A member 4 (Pdia4) and protein disulfide isomerase family A member 6, (Pdia6) (Fig 5C). At both younger-adult and older-adult timepoints, we identified CR-dependent positive regulation of cell survival and longevity factors Kl [32] and transthyretin (Ttr) [33] (Fig 3C), previously unknown to be CR-responsive genes within the hippocampus.

## Discussion

Through unbiased mRNA sequence analysis and targeted NanoString nCounter validation of the CA1 hippocampal region at two distinct timepoints during the adult mouse lifespan, we demonstrate that CR dramatically suppresses age-dependent transcriptional changes. Expression levels of CR-suppressed genes, which are functionally related to intrinsic signaling properties of neurons, were often maintained at levels comparable to those identified in younger mice. In effect, our results show that CR prevents hippocampal CA1 aging at the transcriptional level for hundreds of individual genes. The comparison of CR-dependent transcriptional responses at younger-adult and older-adult timepoints provides valuable insight into the impact diet has on a hippocampal sector involved in learning and memory during a critical duration of adulthood. We posit that CR-mediated repression of age-related transcriptional changes primes the brain for delayed transition from adulthood into senescence. Since the average median wild-type mouse lifespan is approximately 20–26 months, comparison of the described changes in the context of functional assessments at later timepoints would be useful for confirming the magnitude and directionality of CR-mediated gene expression changes and their contribution to successful hippocampal aging throughout the lifespan. Analysis of gene expression changes via RNA-seq, driven by long-term CR in functionally distinct brain areas or hippocampal subregions, including the dentate gyrus, entorhinal cortex, hippocampal CA3 sector, and the subicular complex, would further enhance our understanding of the capacity of CR to suppress brain aging, but are beyond the scope of the present study. Researchers have hypothesized prevention and/or amelioration of hippocampal aging processes and underlying molecular profiles by CR, but to our knowledge, no transcriptional suppression signature of this magnitude has been identified previously.

Specific age-dependent gene expression changes suppressed by CR merit further consideration. In terms of activity-dependent glutamatergic neurotransmission, both Grin2a and Grin2b were downregulated in normal hippocampal CA1 region aging, and these changes were attenuated by CR. Genetic enhancement of Grin2b expression increases NMDA receptor-dependent synaptic plasticity and associated learning and memory functions [34], and hippocampal reductions in Grin2a and Grin2b have been observed in the AD brain, correlating with cognitive decline [35]. We also demonstrate that CR reduces age-dependent downregulation Gria1 mRNA levels, which has been shown to decrease within the hippocampal CA1 region in both in normal aging and in AD [23,36]. Activity-dependent promotion of synaptic strength leading to LTP is thought to require additional insertion of Gria1-containing AMPA receptors in the synaptic membrane, downstream of NMDA receptor activation [37]. Accordingly, we posit that mitigation of age-dependent reductions in NMDA receptor subunits Grin2a and Grin2b and Gria1-containing AMPA receptors following CR could underlie previously observed promotion of LTP throughout aging [30,38]. Furthermore, stress-related Crh and Crhr1 mRNA levels were upregulated by more than 2-fold by normal aging, and CR suppressed age-dependent expression of both of these transcripts to near 5 month old levels. Chronic increases in Crh signaling exacerbates pathology in mouse models of AD [39,40]. Accordingly, we speculate that this suppression signature may confer a protective benefit in the context of blunted stress signaling throughout aging. Our results also support observations demonstrating CR-dependent upregulation of neurotrophic factors Bdnf and Ntf3 following CR [11], further endorsing CR promotion of pro-survival signaling within aging-responsive hippocampal circuits.

Despite observing strikingly different transcriptional responses to CR in 5 and 15 month expression profiles, we identified conserved upregulation of several genes involved in chaperone mediated protein folding and endoplasmic reticulum-mediated calcium buffering, including Calr, Hspa1b, Hspa5, Pdia4, and Pdia6 [41,42]. While these encoded proteins all play essential roles in protein folding and calcium sequestration, Hspa5, also known as glucose regulated protein, 78 kDa (GRP78), is of particular interest, as it binds to the amyloid precursor protein (APP) to subsequently reduce pathological β-amyloid (Aβ) secretion [43] and reduces Aβ neurotoxicity *in vitro* [44]. Our group has also demonstrated that long-term CR reduces Aβ deposition and components of the gamma-secretase complex within the hippocampus of aged female Tg2576 mice [16]. In CR-fed 5 and 15 month old mice, therefore, conserved upregulation of genes with dual roles in proteostasis and calcium buffering, processes thought to contribute to hippocampal CA1 pathological vulnerability, suggests that these CR transcriptional changes likely bestow positive benefits, particularly regarding hippocampal AD pathology prevention.

Notably conserved within the subset of 32 CR-dependent transcriptional changes following CR feeding, we report upregulation of putative anti-aging, neuroprotective genes, Kl and Ttr, which to our knowledge, have not been shown to be CR-responsive previously within the hippocampus. Kl was originally identified as a mutated loss of function gene that accelerated systemic aging [45], and subsequent Kl overexpression demonstrated lifespan extension and reduction of age-dependent insulin resistance [46], as well as preservation of cognitive function and associated synaptic plasticity in an AD mouse model [47]. Brain Kl levels decline with age [48], and Kl-deficient mice display a hippocampal degenerative phenotype [32], although the precise neuronal Kl functions remain unclear. We show that CR endogenously increases Kl mRNA levels in the hippocampal CA1 region in both younger-adult and older-adult mice. We also report conserved increases in Ttr transcript levels at 5 and 15 months of age. Several studies of AD mouse models have suggested that the protein encoded by Ttr may promote pathology resistance by sequestering Aβ to prevent fibril formation and reduce oligomeric cytotoxicity [49,50]. Accordingly, upregulation of Ttr by CR may be a mechanism that confers neuroprotective benefits and AD-like pathology reductions [12,16].

The CR-dependent transcriptional signatures described herein are likely to afford significant benefits in models of neurodegenerative disease, especially AD, and further investigations are planned to probe whether CR activates similar presumed neuroprotective programs within such models. The majority of the human population with AD, however, do not have known mutations associated with their disease. Therefore, AD is believed to arise from gradual age-dependent dysfunction in multiple cellular processes in vulnerable brain regions such as the hippocampal CA1 region. Within this context, studying the influence of CR on brain aging in wild-type mice is an important comparator to normal human aging, relative to transgenic models.

By comparing the influence of CR on the hippocampal CA1 transcriptional profiles of younger-adult and older-adult wild-type mice through total mRNA sequencing and select NanoString nCounter validation, our results demonstrate broad suppression of age-dependent gene expression changes in numerous gene classes. Functional annotation of our mRNA sequencing datasets implicates positive regulation of neuronal processes often deregulated in vulnerable CA1 neurons throughout aging, including activity-dependent synaptic plasticity and calcium signaling. Comparisons at 5 and 15 months of age indicate that CR-dependent transcriptional signatures are highly divergent during aging, with only 32 common transcriptional changes observed. Within conserved changes, however, we identify significant upregulation of several pro-survival genes expected to confer neuroprotective utility, including an enrichment of protein folding and calcium buffering genes. Taken together, these findings provide novel insight into transcriptional signatures underlying CR intervention in hippocampal CA1 aging, while galvanizing the significance of prior investigations on the beneficial influence of CR on brain aging processes. Our findings strongly support the hypothesis that CR globally attenuates brain aging through maintenance of youthful expression signatures and activation of neuroprotective expression changes within the selectively vulnerable hippocampal CA1 region.

## Supporting Information

S1 Table Total differentially expressed genes in normal aging in the hippocampal CA1 region (AL, 15 vs. 5 months). (XLS)

S2 Table Total differentially expressed genes as a function of diet in the aged hippocampal CA1 region (15 months, CR vs. AL). (XLS)

S3 Table Transcriptional changes implicated in IPA predictions of CR-opposed cellular functions during normal aging. (p<0.01, q<0.05).
(DOC)

S4 Table Signature of 882 age-dependent expression changes suppressed by CR. (XLS)

S5 Table Age-dependent transcriptional changes exacerbated by CR. (p<0.01, q<0.05).
(DOC)

S6 Table Total differentially expressed genes as a function of diet in the young hippocampal CA1 region (5 months, CR vs. AL). (XLS)
