# Supplementary material for: Calorie Restriction Suppresses Age-Dependent Hippocampal Transcriptional Signatures
Source: PLoS One. 2015 Jul 29;10(7):e0133923. doi: 10.1371/journal.pone.0133923 (PMC4519125; doi:10.1371/journal.pone.0133923)
Supplement: S3 Table — (p<0.01, q<0.05). (DOC) [file pone.0133923.s003.doc]

**Supplemental Table 3.**

Transcriptional changes implicated in IPA predictions of CR-opposed cellular functions throughout aging.

| **Comparison** | **Cellular function** | **z-score** | **Upregulated genes** | **Downregulated genes** |
| --- | --- | --- | --- | --- |
| AL, 15 vs. 5mo | Growth of neurites | 2.3 | Bcl2, Cables1, Dcx, Efnb2, Fn1, Gap43, Hdgfrp3, Hgf, Igsf9, Kitlg, Kndc1, L1cam, Limk1, Lrrk2, Ngef, Ngfr, Npas4, Nrg1, Plxna3, Pparg, Ptprf, Reln, Sema3a, Sema3f, Sh2d3c, Tiam1, Wnt5a | Cnr1, Efnb3, Enpp2, Ephb3, Gfap, Gfra1, Kit, Mag, Myh9, Neu4, Nf2, Nfia, Nrp1, Nsmf, Plxna4, Prokr2, Robo1, Sema5a, Sstr3, Trpc5, Zbtb18 |
| AL, 15 vs. 5mo | Growth of axons | 2.1 | Bcl2, Dcx, Gap43, L1cam, Limk1, Lrrk2, Ngef, Ngfr, Nrg1, Plxna3, Sema3a, Sema3f, Sh2d3c, Wnt5a | Efnb3, Enpp2, Ephb3, Gfap, Gfra1, Mag, Nfia, Nrp1, Nsmf, Plxna4, Prokr2, Robo1, Sema5a, Zbtb18 |
| AL, 15 vs. 5mo | Long-term depression | -2.6 | Camk4 | Cnr1, Drd5, Gria1, Gria2, Grm1, Grm5, Itpr1, Pebp1, Prkca, Prkcg, Ryr3 |
| 15mo, CR vs. AL | Formation of neurites | 2.2 | Nfia, Nfib, Nrp1, Ppp1r9b, Snca | Cit, Dcx, L1cam, Sema3a |
| 15mo, CR vs. AL | Quantity of monoamines | 2.2 | Fth1, Gfra1, Gria1, Htr1a, Snca, Tnfrsf25 | Gap43, Htr2c, Oprk1 |
| 15mo, CR vs. AL | Synaptic transmission | 2.0 | Gria1, Grin2a, Grin2b, Htr1a, Htr4, Nr3c2, Prkcg, Shank3, Snca | Casr, Fgf12, Ntsr1, Unc13c |
| 15mo, CR vs. AL | Quantity of neurons | 2.0 | Bdnf, Crlf1, Emx2, Fgfr1, Fth1, Gal, Gfra1, Htr4, Nde1, Neurod1, Neurod2, Nrp2, Nsmf, Ntf3, Snca, Tgfa, Tgfb2, Trpa1, Zbtb18 | Cux1, Dfnb31, Dusp1, Egr2, Gfra2, L1cam, Met, Neurod4, Sema3a, Tbr1 |

(p<0.01, q<0.05)
