# Supplementary material for: Calorie Restriction Suppresses Age-Dependent Hippocampal Transcriptional Signatures
Source: PLoS One. 2015 Jul 29;10(7):e0133923. doi: 10.1371/journal.pone.0133923 (PMC4519125; doi:10.1371/journal.pone.0133923)
Supplement: S5 Table — (p<0.01, q<0.05). (DOC) [file pone.0133923.s005.doc]

Supplemental Table 5.

Age-dependent transcriptional changes exacerbated by CR.

|  | | **Normalized expression levels (FPKM)** | | |
| --- | --- | --- | --- | --- |
| **Gene ID** | **Gene Name** | **AL, 5mo.** | **AL, 15mo.** | **CR, 15mo.** |
| Agxt2l1 | Alanine-glyoxylate aminotransferase 2-like 1 | 0.73 | 4.28 | 7.30 |
| Calb2 | Calbindin 2 | 15.41 | 29.15 | 48.81 |
| Col8a2 | Collagen, type VIII, alpha 2 | 0.95 | 0.45 | 0.20 |
| Rtp1 | Receptor transporter protein 1 | 0.54 | 1.37 | 2.30 |
| Stab2 | Stabilin 2 | 0.16 | 0.58 | 1.27 |

(p<0.01, q<0.05)
